# Supplementary figures and images for: Development and validation of the health education demand scale for HPV infected patients based on KANO model
Source: PLoS One. 2025 Jan 3;20(1):e0309630. doi: 10.1371/journal.pone.0309630 (PMC11698313; doi:10.1371/journal.pone.0309630)

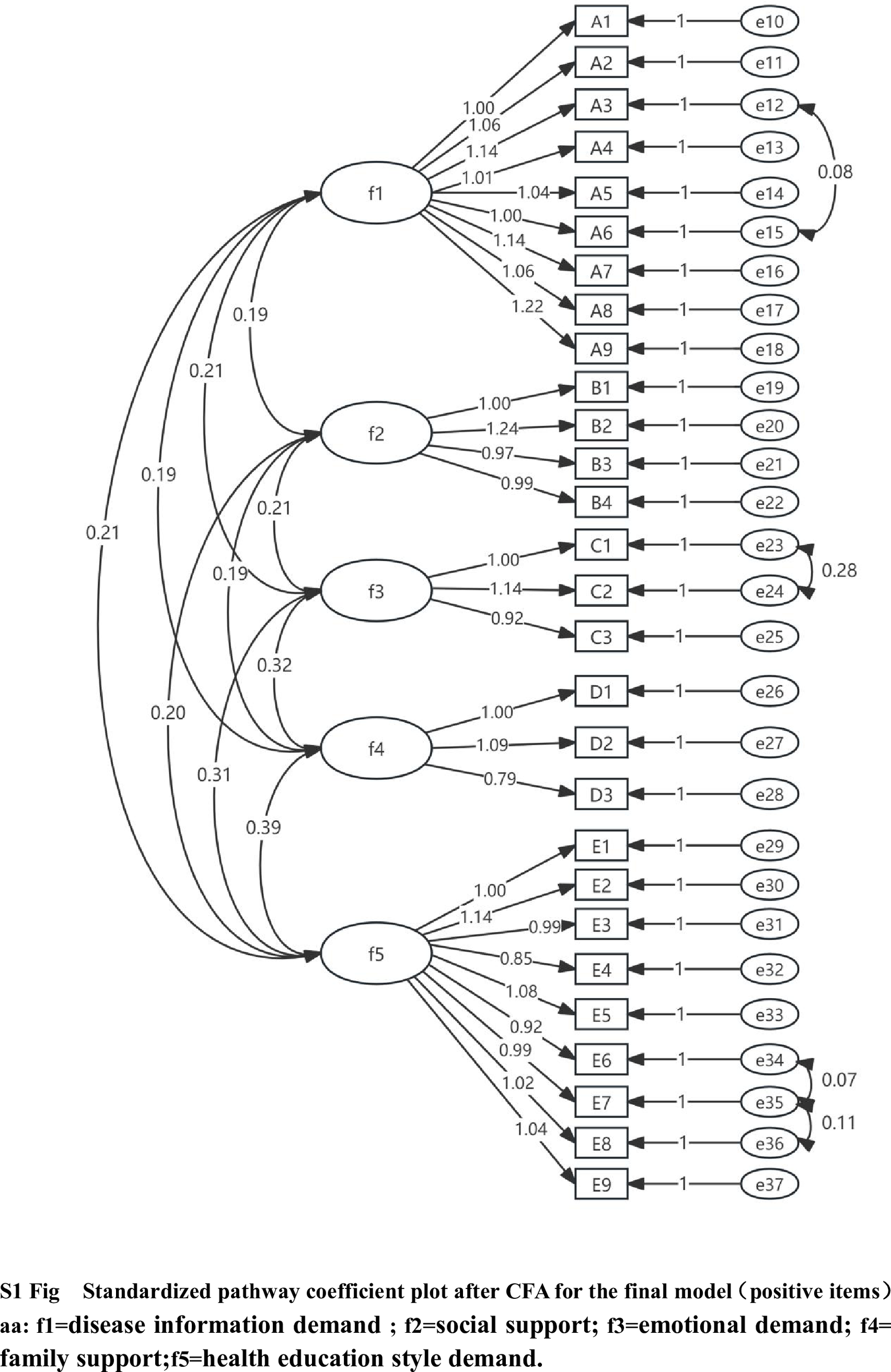

Supplement: S1 Fig — aa: fl = disease information demand; f2 = social support; f3 = emotional demand; f4 = family support; f5 = health education style demand. (TIF) [file pone.0309630.s001.tif]
