# Supplementary material for: Development and validation of the health education demand scale for HPV infected patients based on KANO model
Source: PLoS One. 2025 Jan 3;20(1):e0309630. doi: 10.1371/journal.pone.0309630 (PMC11698313; doi:10.1371/journal.pone.0309630)
Supplement: S1 File — (DOCX) [file pone.0309630.s003.docx]

**The health education demand scale for HPV infected patients based on KANO model**

Dear patient friends,

Hello! We are a VIP clinic research team of West China Second Hospital of Sichuan University. We are conducting a survey on the information needs of patients with HPV infection. In order for you to have a better medical experience, we hope to understand your real needs in HPV-related disease information, psychological and social aspects through this study.

Participation in this study is entirely voluntary and you can opt out at any time. At the same time, your personal information will be kept strictly confidential, the content of your answer is intended for research, and the data will not be fed back to any other person or organization except the researcher. We will keep the information you fill in confidential and hope to get your support. Please complete the questionnaire and submit it to the investigator. Thank you for your support and cooperation!

***Part one Sample characteristics***

1. Age: ______

2. The family long-term residence: ①village ②suburb ③City ④Other

3. The frequency of your cervical cancer screening is: ① never ② once every 3 years or more ③ every 2 years ④ once a year ⑤ Multiple times a year

4. Marital status: ① Unmarried ② divorced ③ widowed ④ married

5. You and your spouse's lifestyle: ① cohabitation ② Live apart ③ other

6. You and your spouse sexual frequency: ①1-3 times a month ②1-3 times a week ③4-6 times a week ④≥7 times a week

7. Whether you have a sexual partner: ① yes ② no

8. Frequency of sexual intercourse with sexual partners: ①1-3 times a month ②1-3 times a week ③4-6 times a week ④≥7 times a week

9. Your age of first sexual intercourse: ① never ② < 16 ③16-25 ④26-35 ⑤ > 35

10. Frequency of oral contraceptives: ① < 1 time a month ②1-3 times a month ③≥3 times a month

11. Your number of pregnancies: ① never ② 1 ③2 ④≥3

12. From the past to the present, the number of your sexual partners: ①1 ②2 ③≥3

***Part two Formal scale***

Please provide your answer by using the rating below: Check the number in the box that corresponds to your answer.

5 –like

4 – take for granted

3 – don't care

2 – can tolerate

1 – don't like

| **Disease information demand** | **1** | **2** | **3** | **4** | **5** |
| --- | --- | --- | --- | --- | --- |
| 1. How do you feel if you are provided health guidance on HPV prevention? |  |  |  |  |  |
| 2. How do you feel if you are provided with information about HPV vaccine (type, vaccination)? |  |  |  |  |  |
| 3. How do you feel if you are provided about health education on HPV screening (HPV typing, TCT)? |  |  |  |  |  |
| 4. How do you feel if you are provided about providing HPV-related screening and prevention guidance to sexual partners? |  |  |  |  |  |
| 5. How do you feel if you are provided relevant knowledge about the relationship between HPV infection and cervical lesions? |  |  |  |  |  |
| 6. How do you feel if you are provided with guidance for HPV infection related tests (colposcopy, biopsy)? |  |  |  |  |  |
| 7. For HPV, how do you feel if you are told the method and time of the review? |  |  |  |  |  |
| 8. How do you feel if you are provided information about the treatment for yourself and your sexual partner? |  |  |  |  |  |
| 9. How do you feel if you are told about the prognosis (quality of life, recurrence)? |  |  |  |  |  |
| **Social support** |  |  |  |  |  |
| 10. For HPV, how do you feel if you are told about self-care guidance and precautions? |  |  |  |  |  |
| 11. How do you feel if we instruct you to avoid risk factors related to recurrence? |  |  |  |  |  |
| 12. How do you feel if you are informed about the latest developments in the treatment of HPV infection? |  |  |  |  |  |
| 13. How do you feel if you are provided a way to understand doctors' areas of expertise in diseases? |  |  |  |  |  |
| **Emotional demand** |  |  |  |  |  |
| 14. How do you feel if you are provided about health education on fertility possibilities and reproductive decision-making? |  |  |  |  |  |
| 15.How do you feel if you are provided places and platforms for expressing emotional states? |  |  |  |  |  |
| 16.How do you feel if professional psychological counseling and support (individual psychological counseling, group psychotherapy) are provided? |  |  |  |  |  |
| **Family support** |  |  |  |  |  |
| 17. How do you feel if the family members are informed to participate in the management of the disease together? |  |  |  |  |  |
| 18. How do you feel if family members are allowed to accompany them when necessary? |  |  |  |  |  |
| 19. How do you feel if we help patients maintain and build intimate relationships with their partners? |  |  |  |  |  |
| **Health education style demand** |  |  |  |  |  |
| 20. How do you feel if a health education environment with warm environment, suitable temperature and teaching conditions is provided? |  |  |  |  |  |
| 21. How do you feel if you are informed the specific content or time of health education through SMS or push? |  |  |  |  |  |
| 22.How do you feel if HPV-related health education knowledge manuals are distributed? |  |  |  |  |  |
| 23. How do you feel if doctors participate in HPV-related knowledge education? |  |  |  |  |  |
| 24. How do you feel about the case management platform for patients with HPV infection? |  |  |  |  |  |
| 25. How do you feel about online HPV disease-related health consultation? |  |  |  |  |  |
| 26. How do you feel if an article about HPV infection is pushed online? |  |  |  |  |  |
| 27. How do you feel if a short health education video related to HPV infection is pushed online? |  |  |  |  |  |
| 28. How do you feel if we provide online psychological counseling and communication? |  |  |  |  |  |

Above are the positive items of the scale.
