# Supplementary material for: Development and validation of the health education demand scale for HPV infected patients based on KANO model
Source: PLoS One. 2025 Jan 3;20(1):e0309630. doi: 10.1371/journal.pone.0309630 (PMC11698313; doi:10.1371/journal.pone.0309630)
Supplement: Attachment — Submitted filename: Response to Reviewers.docx [file pone.0309630.s004.docx]

**Response to Reviewers**

**Dear** Dr. **Nicola Serra and reviewers:**

**Thank you for your letter and for the insightful comments concerning our manuscript entitled “Development and validation of the health education demand scale for HPV infected patients based on KANO model” (Manuscript ID PONE-D-24-32314). Those comments are all valuable and helpful for revising and improving our paper.**

**We have studied comments carefully and have made correction which we hope meet with approval. Revised portions are marked in red in the manuscript.**

**The main corrections in the paper and respond to the reviewer’s comments are as following:**

****Journal Requirements:****

**When submitting your revision, we need you to address these additional requirements.**

1.**Please ensure that your manuscript meets PLOS ONE's style requirements, including those for file naming.**

****Response：**We have changed the naming of the file as requested by PLOS ONE.Thanks.**

2. **PLOS requires an ORCID iD for the corresponding author in Editorial Manager on papers submitted after December 6th, 2016. Please ensure that you have an ORCID iD and that it is validated in Editorial Manager.**

****Response：**We have applied ORCID iD and validated in Editorial Manager. Thanks.**

3. **We notice that your supplementary figures are uploaded with the file type 'Figure'. Please amend the file type to 'Supporting Information'. Please ensure that each Supporting Information file has a legend listed in the manuscript after the references list.**

****Response :**We have changed the file type "Figure" to "Supporting Information" and ensure that each Supporting Information file has a legend listed in the manuscript after the references list.**

**Additional Editor Comments:**

**1. Statistical analysis section.**

**This section is the key to understanding the statistical analyses performed in the manuscript. Authors should specify how numerical and qualitative data were described. For example, in the case of numerical data, authors should report, the numerical data were reported as mean ad standard deviation, or median and interquartile range [Q1, Q3], in the case of non-normally distributed data. Furthermore, they should report which statistical tests were used and for which analyses. Finally, they should conclude the paragraph by indicating what level of p-value was chosen to consider a test significant, e.g. All tests with p-value <0.05 were considered as significant.**

****Responses：****

**Thanks to the editors for their valuable suggestions. The description of quantitative and qualitative data in statistical analysis has been supplemented in the data analysis section of the paper. Qualitative data is expressed as frequency and component ratio (%), and quantitative data conforming to normal distribution is expressed as mean ± standard deviation. P<0.05 was considered statistically significant.**

2.**A brief description of Tables 3 and 4 in the results should be made**

****Responses：****

**Thanks for the valuable suggestions provided by the editor. We have made a brief description of table 3 and table 4 in the paper. Table 3 is the factor analysis result of the positive items, table 4 is the factor analysis result of the reverse items, and we finally focus on the result of table 3 (factor analysis result of the positive items).**

3. **Sample and setting section. The authors should better describe how they estimated the sample size, including the values of the parameters used for the calculation.**

****Responses：****

**Thanks for the editor's valuable suggestions. We have explained the sample size in the paper. According to Kendall's scale preparation sample size setting rule, the sample size is set to 5-10 times of the scale items, and the factor analysis sample size is set to 10-20 times of the scale items, and 10% sample shedding rate is also considered.**

**Reviewers' comments:**

2. **Has the statistical analysis been performed appropriately and rigorously?**

****Response :****The data was analyzed using IBM SPSS Statistical window (version 20.0), and IBM SPSS AMOS (version 23.0). Our data analysis strictly conforms to the steps of the scale compilation, and the collected data are analyzed by project analysis, retest analysis and reliability and validity test, among which the validity test included content validity and structure validity.

****Reviewer #1: GENERAL COMMENT:**

The manuscript aimed to develop and validate a health education needs assessment for patients with HPV infection using the KANO model. The final product is a fascinating picture of the types of health education needs and the influencing factor of patients with HPV infection to create personalized health education programs.
I only suggest a few tips to improve the quality of the manuscript, such as revising the English language and emphasizing some topics.

**INTRODUCTION:**
Human papillpn virus (HPV) is a virus with multiple genotypes, which is closely related to the degree of human reproductive system lesions and the development of malignant tumors.
Please correct the virus name.**

**It seems that your introduction is too short. Please emphasize more topics, such as the impact of HPV on cervical lesions and the socio-demographic profile of people with sexually transmitted infections.**

****Response：****

**Thanks for the reviewer's suggestions. We have changed the virus name in the article, and we have added the impact of HPV on cervical lesions and the socio-demographic profile of people with sexually transmitted infections.**

****Reviewer #2: GENERAL COMMENT:**
The manuscript contributes to understanding the HPV health education needs from the perspective of the disease, the patient's family, and social, environmental, psychological and other internal and external systems. I believe that the workflow is well-designed and well-structured, but I would like to raise some issues you can find below.
I suggest English language editing.

**INTRODUCTION:**
Page 1: “Human papillpn virus”
Please, correct the spelling.**

****Response：****

**Sorry for the spelling error, the spelling of HPV virus has been corrected. Thanks.**

**Page 1: “..degree of human reproductive system lesions..”**

**What do you mean by this expression?**

****Response：****

**We are very grateful to the reviewer for raising such questions, and we have revised the expression to mean that HPV infection is related to the severity of cervical lesions.**

**Page 1: “…cervical cancer is also gradually getting younger...”
I understand that you meant that the age of patients suffering from cervical cancer is getting lower, but I believe you should rephrase the sentence. It is not clear enough as it is.
Moreover, please put a reference attesting to this data.**

****Response：****

**Thanks for the reviewer's suggestion, we have modified the expression to make it better understood. In addition, we also supplement the relevant evidence. Thanks.**
