# Supplementary material for: Development and validation of the health education demand scale for HPV infected patients based on KANO model
Source: PLoS One. 2025 Jan 3;20(1):e0309630. doi: 10.1371/journal.pone.0309630 (PMC11698313; doi:10.1371/journal.pone.0309630)
Supplement: Attachment — Submitted filename: Response to Reviewers(2).docx [file pone.0309630.s005.docx]

**Response to Reviewers**

**Dear** Dr. **Nicola Serra and reviewers:**

**Thank you again for your letter and insightful comments on our article entitled "Development and Validation of the KANO Model-based Health Education Needs Scale for Patients with HPV Infection" (manuscript ID PONE-D-24-32314R1). All these opinions are valuable for our paper to be improved again.**

**We have read the proposed changes again and made corrections, hoping for your approval. The revised parts are highlighted in red in the manuscript.**

**The main changes and responses to the reviewer's comments are as follows:**

****Journal Requirements:****

**Please review your reference list to ensure that it is complete and correct. If you have cited papers that have been retracted, please include the rationale for doing so in the manuscript text, or remove these references and replace them with relevant current references. Any changes to the reference list should be mentioned in the rebuttal letter that accompanies your revised manuscript. If you need to cite a retracted article, indicate the article’s retracted status in the References list and also include a citation and full reference for the retraction notice.**

****Response：**Thank you for your valuable suggestions. We have reviewed our reference list and ensure that it is complete and correct. We have carefully reviewed all the citations, and none have been retracted. In addition, PLOS uses the reference style outlined by the International Committee of Medical Journal Editors (ICMJE), also referred to as the “Vancouver” style.The references we cite meet this format requirement.**

**Reviewers' comments:**

****Reviewer #2: The authors have accurately followed the suggestions. However, I would like to make some notes.****

**INTRODUCTION:**

**Page 1: “The higher infection rate of HPV, the more serious of cervical precancerous lesions.”**

**Can I suggest rephrasing the sentence? It would be clearer to exchange the order in something like: “More severe precancerous lesions are associated with higher rates of HPV infection”**

**Page 1: “Additionally, the age-specific distribution of HPV infection showed a bimodal curve, and the trend of younger age was obvious”**

**I believe that you should better explain this observation. What do you mean specifically with “the trend of younger age was obvious”? It seams quite a vague comment.**

****Response :**Thank you for your valuable suggestions. For the expression of “The higher infection rate of HPV, the more serious of cervical precancerous lesions.”, we have changed the expression. For the meaning of “the trend of younger age was obvious”, we also made changes to make it clearer.**
